# Protein hyperproduction in fungi by design

**DOI:** 10.1007/s00253-018-9265-1

**Published:** 2018-08-04

**Authors:** Scott E. Baker

**Affiliations:** 1 Department of Energy Joint BioEnergy Institute, Emeryville, CA 94608 USA; 2 Biosystems Design and Simulation Group, Environmental Molecular Sciences Division, Earth and Biological Sciences Directorate, Pacific Northwest National Laboratory, Richland, WA 99352 USA

**Keywords:** Enzyme, Protein, Hyperproduction, Secretion, Biodesign, Fungi, Biotechnology

## Abstract

The secretion of enzymes used by fungi to digest their environment has been exploited by humans for centuries for food and beverage production. More than a century after the first biotechnology patent, we know that the enzyme cocktails secreted by these amazing organisms have tremendous use across a number of industrial processes. Secreting the maximum titer of enzymes is critical to the economic feasibility of these processes. Traditional mutagenesis and screening approaches have generated the vast majority of strains used by industry for the production of enzymes. Until the emergence of economical next generation DNA sequencing platforms, the majority of the genes mutated in these screens remained uncharacterized at the sequence level. In addition, mutagenesis comes with a cost to an organism’s fitness, making tractable rational strain design approaches an attractive alternative. As an alternative to traditional mutagenesis and screening, controlled manipulation of multiple genes involved in processes that impact the ability of a fungus to sense its environment, regulate transcription of enzyme-encoding genes, and efficiently secrete these proteins will allow for rational design of improved fungal protein production strains.

## Introduction

Fungi are constantly digesting their environment, secreting degradative enzymes, and absorbing the building block nutrients that are released. For centuries, humans have endeavored to harness the secreted enzyme activity, largely for production of food and beverage products such as soy sauce or sake (Abe and Gomi 2008; Baker and Bennett 2007; Machida et al. 2008). However, as the diversity of characterized enzymatic activities grows, so too do the potential uses (reviewed in Østergaard and Olsen 2011). Since the issuance of the first biotechnology patent in 1894 focused on production of starch saccharification enzymes from *Aspergillus oryzae* (Takamine 1894), fungi have been used to understand the basic biology of enzymes and to develop systems for their industrial production for use in a variety of applications. For example, since World War II, pioneering research and development have been performed in *Trichoderma reesei*, from basic research that includes the elucidation of the components for the cellulose degradation enzyme system (Reese 1976) to applied research that includes the development of strains such as *T*. *reesei* RUT-C30 that are the parents of strains used by the industry to produce enzyme cocktails for lignocellulosic biofuel production (Peterson and Nevalainen 2012). In the case of lignocellulosic biofuel and bioproduct production, where an enzyme or enzyme cocktail rather than the enzymatic process is being sold, a key factor for economic viability of enzyme sales is the cost and efficiency of enzyme production (Klein-Marcuschamer et al. 2012).

Over the last century, traditional forward genetic mutagenesis and screening methods have been utilized to generate strains with increased titer, rates, and yields of desired secreted enzymes. For example, *Aspergillus niger* strains with improved production of multiple types of enzymes, including glucoamylase (Armbruster 1961; Hu et al. 2017; Nevalainen 1981; Tahoun 1993) and *T*. *reesei* strains that produce high titers of cellulase (Mandels et al. 1971; Montenecourt and Eveleigh 1977; Peterson and Nevalainen 2012), have been generated by a variety of mutagenesis and screening regimes. With the continued industrialization and decreasing cost of DNA sequencing, it is now possible to “resequence” these mutant strains, identify mutations of interest, and assess mutations in a “clean” genetic background for their effect on enzyme secretion using reverse genetic methods (Baker 2009; Baker and Bredeweg 2016; Ivanova et al. 2017; Koike et al. 2013; Le Crom et al. 2009; Lichius et al. 2015; Nitta et al. 2012; Vitikainen et al. 2010). In this way, a number of mutations have been characterized that have led to increased enzyme secretion (Nitta et al. 2012; Pei et al. 2015). Derivatives of mutagenized strains continue to be developed and used by the industry for production of enzymes (Schuster et al. 2002; van Dijck et al. 2003). Although mutagenesis is effective at generating strains that secrete significant titers of enzymes, strain improvement often comes with collateral genome damage. For example, in the case of *T*. *reesei*, the quest for strains hyperproducing cellulases also led to cellulose-negative strains (Druzhinina et al. 2006; Ivanova et al. 2017; Lichius et al. 2015; Torigoi et al. 1996). Moreover, it is only within the last decade that the sexual cycle of *T*. *reesei* was described and the possibility of classical genetic strategies for understanding and improving protein hyper-production explored (Jourdier et al. 2017; Kuck and Bohm 2013; Li et al. 2016; Linke et al. 2015; Seidl and Seiboth 2010; Tisch et al. 2017).

Beyond industrial biotechnology enzyme and small molecule production hosts, yeast and filamentous fungi are well studied as model systems for a number of biological processes that include, but are not limited to, protein secretion, cell signaling, cell morphology, and small molecule transport. Approaches from a breadth of biological disciplines, such as genetics, genomics, cell biology, physiology, molecular biology, and biochemistry have been used to understand the biological processes that underlie the fungal lifestyle. Decades of basic and applied fungal research spanning a breadth of methods has generated a knowledgebase that makes it possible to rationally design hypersecreting fungal enzyme production hosts.

**Fig. 1:**
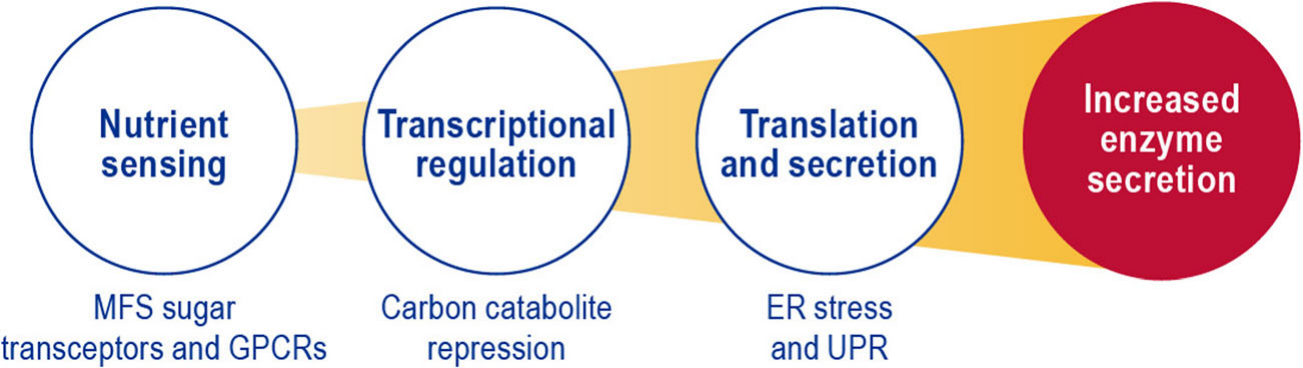
Manipulation of genes that encode proteins involved in regulating nutrient sensing, transcription, translation, and secretion is key for rational design of fungal lignocellulosic deconstruction enzyme hypersecretors

This mini review focuses on a subset of biological processes involved in ascomycete production of carbohydrate-active enzymes (CAZymes). Enzymatic deconstruction of various plant biomass components is considered a critical step in the production of lignocellulosic biofuels, and there is a vast literature on the genetics, biochemistry, cell biology, and regulation of CAZyme secretion from ascomycetes. In the following sections, I describe three different biological processes that contribute to filamentous fungal enzyme secretion: (1) nutrient sensing; (2) transcriptional regulation, and (3) translation and secretion (Fig. 1). I also overview recent research that uses a rational design strategy for a filamentous fungal hypersecreting enzyme production host that incorporates manipulating genes whose products are involved in these biological processes.

## Nutrient sensing

Fungi must balance the need to produce enzymes to digest complex substrates and provide building block nutrients with the level of available building block nutrients for absorption; the expense of secreting an enzyme must be balanced with nutritional return on the biosynthetic investment and growth rate. The first step in managing these trade-offs is to be able to sense the concentration of nutrients available for absorption. To accomplish this, fungi exploit a breadth of sensors that are the first step in “seeing” environmental conditions. Blinding the fungus to its nutritional landscape is one way to induce high expression of digestive enzymes relevant to industrial applications. In order to “blind” a fungus, deletion of the appropriate sensing proteins is critical. Over the last several decades, researchers have done much to increase this knowledge.

Early hints at the exquisite control exerted by filamentous fungi on absorption of sugars came from a study of a pyruvate carboxylase mutant of *Aspergillus nidulans*. In this mutant strain, growth on glucose resulted in secretion of pyruvate while growth on glucose in the presence of acetate resulted in pyruvate not being secreted, indicating that glucose was not being utilized by the organism (Romano and Kornberg 1969). This regulation of glucose uptake illustrated feedback from central metabolism to regulate sugar uptake and launched subsequent studies into sugar transport systems. A major early finding indicated distinct systems for uptake of specific sugars in *A*. *nidulans* and *Neurospora crassa* (Mark and Romano 1971; Scarborough 1970). Moreover, it was shown that both high- and low-affinity uptake systems exist for glucose and other sugars in these and other fungi (MacCabe et al. 2003; Schneider and Wiley 1971; Torres et al. 1996; Wang et al. 2017). Thus, it is apparent that fungi “see” the nutrient environment around them and are able to respond with appropriate enzymes and transporters needed for generation, uptake, and metabolism of these building blocks.

In *N*. *crassa*, a gene originally identified for its role in conidiation, *rco-3*, was cloned and sequenced and found to encode a protein with significant homology to known glucose transporters (Madi et al. 1997). Experimental results indicated that its function may be more complex than simply glucose transport. In experiments with 2-deoxy-D-glucose (2DG), a glucose analog that inhibits glycolosis and is used in studies of carbon catabolite repression, mutant *rco-3* strains exhibited glucose transport properties consistent with defects in the glucose repression system rather than a defect in a single glucose transporter (Ebbole 1998; Madi et al. 1997). Recently, other putative sugar transporters tied to carbon catabolite repression have been identified in *N*. *crassa*, *A*. *nidulans* and *A*. *niger* (Dos Reis et al. 2017; Reilly et al. 2018; Wang et al. 2017). In some instances, predicted transporters connected with activation of signaling networks have been called “transceptors” because they embody structure and/or behavior associated with transporters and receptors (Van Dijck et al. 2017).

G-protein-coupled receptors (GPCRs) are located at the plasma membrane, have seven transmembrane domains, and are centrally involved in environmental sensing and signaling. Several fungal GPCRs have been characterized, a subset of which are involved in sugar sensing (Xue et al. 2008). Both *N*. *crassa* and *A*. *nidulans* have GPCRs that have been identified and characterized as being involved in carbon source sensing (Brown et al. 2015; Li and Borkovich 2006). When GPCRs are activated, adenylate cyclase increases levels of cyclic AMP, which in turn activates protein kinase A (PKA) downstream signaling cascades. One target of this activity is carbon catabolite repression; molecular genetic studies in *A*. *nidulans* and *T*. *reesei* indicate that PKA influences expression of hydrolases (de Assis et al. 2015; Schuster et al. 2012).

## Transcriptional regulation

GPCRs and sugar transporters/transceptors are the first line of environmental sensing feeding into the signaling networks that regulate gene expression and protein secretion. While the mechanisms that exquisitely regulate the carbon catabolite repression system and secretion of lignocellulosic deconstruction enzymes of fungi can differ, some elements of the core control system players are conserved (Benocci et al. 2017; Klaubauf et al. 2014). The gene encoding the master controller for carbon catabolite repression was initially discovered in *Saccharomyces cerevisiae* and named *mig1* (multicopy inhibitor of galactose promoter) (Mercado et al. 1991; Nehlin et al. 1991; Nehlin and Ronne 1990). Overexpression of *mig1* represses carbon catabolism, while deletion of *mig1* predictably affects glucose repression (Nehlin and Ronne 1990). Additional work in *S*. *cerevisiae* and other yeasts of the *Saccharomycotina* has shown that the kinase Snf1p plays an important role in Mig1p activity via phosphorylation (Matsuzawa et al. 2012; Rippert et al. 2017; Treitel et al. 1998).

In filamentous ascomycetes, the ortholog of *mig1* is named *creA* (*Aspergillus*) or *cr*e*1* (*Trichoderma* and *Neurospora*). *Aspergillus nidulans creA* was originally identified by suppressor screens in a nitrogen metabolite repression mutant background (*areA*^*−*^*)* (Arst and Cove 1973). *creA* was later cloned, and orthologues to *creA* have been identified in several other fungi (Cepeda-García et al. 2014; de la Serna et al. 1999; Dowzer and Kelly 1989; Drysdale et al. 1993; Ilmen et al. 1996; Jekosch and Kück 2000; Liu et al. 2013; Tudzynski et al. 2000; Vautard et al. 1999; Wang et al. 2015). Recent research on *A*. *niger* indicates that sequential uptake of different sugar monomers into the cell is regulated separately from metabolism and not by *creA* (Mäkelä et al. 2018).

In the RUT-C30 lineage of *T*. *reesei*, *cre1* is truncated with the mutation occurring in the last stage of mutagenesis on 2DG, highlighting the central role of CRE1 in carbon catabolite repression (Ilmen et al. 1996; Le Crom et al. 2009; Montenecourt and Eveleigh 1977). Subsequent studies indicated that the CRE1 truncation in RUT-C30 (*cre1–1*) and a complete deletion of *cre1* had identical phenotypes, carbon catabolite derepression, and increased hydrolysis enzyme secretion (Nakari-Setala et al. 2009). Post-translational modifications are known to play an important role in *cre1* function; phosphorylation impacts DNA binding in multiple ways depending on the organism (Cziferszky et al. 2002; Vautard-Mey and Fevre 2000). The isolation of *creB*, a predicted deubiquitination enzyme as a suppressor of *creA*, points toward a role of ubiquitination in regulation of CREA/1 function although a detailed mechanism remains unknown. Furthermore, F-box proteins that are part of the complex that comprise the SCF family of E3 ubiquitin ligases (Skowyra et al. 1997) interact genetically with *creA/1* (Colabardini et al. 2012; Jonkers and Rep 2009).

## Translation and secretion

The protein secretion pathway of yeast and filamentous fungi is of high interest as a target for modification in order to increase secretion of proteins. Fungal protein secretion has been explored at all stages, from protein targeting to ER to secretion and subsequent degradation by proteases with the goal of improving titer, rate, and yield of target proteins. The cellular response to ER stress, often referred to as the unfolded protein response or UPR, plays an important role in protein secretion inducing the expression of chaperones and other proteins that aid in folding and protein trafficking efficiency (Malavazi et al. 2014; Mori 2015; Nawkar et al. 2018; Smith and Wilkinson 2017). Activation of the unfolded protein response or UPR pathway is highly conserved and has been well described in a number of eukaryotic systems (Malavazi et al. 2014; Mori 2015; Nawkar et al. 2018; Smith and Wilkinson 2017).

The UPR has been extensively studied in *S*. *cerevisiae*, where various screens identified UPR genes (Mori 2015; Mori et al. 1996). A transcription factor, Hac1p, was shown to be spliced by an unconventional mechanism involving Ire1p (Cox and Walter 1996; Kawahara et al. 1997). This mechanism is conserved in other yeast, filamentous fungi, and other eukaryotes (Guerfal et al. 2010; Hooks and Griffiths-Jones 2011; Mulder et al. 2004; Saloheimo et al. 2003; Whyteside et al. 2011). Once activated, the UPR leads to increased expression of proteins involved in protein folding. In addition, the UPR is known to be involved in ER-associated degradation (ERAD) (Travers et al. 2000) and repression under secretion stress (RESS), which balance ER stress by degrading unfolded proteins and repressing expression of secreted proteins respectively (Pakula et al. 2003). It stands to reason that overexpression of an activated HAC1/A would have a positive impact on protein secretion, producing properly folded proteins while decreasing stress on the ER. This has been demonstrated in a variety of systems, often for heterologous protein production with inducible expression of activated HAC1/A being more effective than constitutive expression (Carvalho et al. 2012; Guerfal et al. 2010; Valkonen et al. 2003a; Valkonen et al. 2003b; Wu et al. 2017). While induction of cellulase gene transcription is not HAC1 or IRE1 dependent, deletion of *hac-1* from *N*. *crassa* results in significantly reduced growth when cellulose is the carbon source (Fan et al. 2015; Montenegro-Montero et al. 2015). Furthermore, a number transcription factors downstream of HAC1 are involved in regulating lignocellulosic deconstruction enzyme-encoding genes, indicating a complex regulatory network influenced by ER stress and the UPR (Fan et al. 2015). Interestingly, the low cellulase production in *hac-1* mutants is suppressed by mutations in sterol regulatory element-binding proteins (SREBPs) demonstrating a connection between the UPR and sterol and lipid metabolism (Qin et al. 2017; Reilly et al. 2015; Volmer and Ron 2015).

## Rational design

Rational design of fungal lignocellulosic deconstruction enzyme hypersecretors should consider nutrient sensing, transcription, translation, and secretion. The availability of tools for genetic manipulation is critical to the rational design of fungal production strains. Approaches for developing transformation systems in filamentous fungi are well established (reviewed in Li et al. 2017a). Both gene deletion and overexpression are critical elements of strain design. In the case of gene overexpression, control of transcription is critical, and development of finely tuned regulatory systems has, for example, been demonstrated for cellulase expression in *N*. *crassa* (Matsu-Ura et al. 2018).

*Penicillium oxalicum* has been a target organism for rational design of lignocellulosic biomass deconstruction enzyme production (Li et al. 2017b). Initially, traditional mutagenesis and screening methods were used to developed high-protein production strains. Initial strains developed have largely manipulated transcriptional regulators, including *creA*, and in subsequent work have generated an activated Xylanase regulator 1 (called *xlnR* (A871V) as well as a chimeric *clrB-xlnR* (A871B) (Derntl et al. 2013; Gao et al. 2017a; Gao et al. 2017b; Li et al. 2015; Yao et al. 2015). Similar combinatorial genetics have been developed in *Myceliophthora thermophile*, a thermophilic ascomycete. In this case, a CRISPR/Cas9 genome editing approach was utilized to combine mutations in genes whose products are involved in carbon catabolite repression, ER stress, and proteolysis (Liu et al. 2017). Future rational design of protein hyperproduction strains should combine nutrient sensing, transcriptional regulation, translation, and secretion (Fig. 1). Genetic manipulation of these processes has been shown to increase protein production. High-level transcriptional regulators (such as CREA/1 and HAC1/A) sugar transceptors and GPCRs as well as their downstream regulatory and signaling cascade proteins are potential targets for genetic manipulation for rational design of protein hyperproducers. A combinatorial approach to identifying synergistic interactions between deletion and controlled overexpression of these genes has the potential to yield highly productive strains with a minimum of non-productive phenotypes.

## Conclusions

Fungi are amazing producers and secretors of enzymes: it is what they do to grow. These digestive enzymes cover a breadth of potential substrates: plant biomass, fungal biomass, proteinaceous substrates, and many others. The enzymes secreted to digest these materials have a huge range of applications for a variety of industries (Østergaard and Olsen 2011). Thus, the repertoire of digestive enzyme activities in combination with an ability to secrete a high titer of protein, make these organisms industrially intriguing. With our current knowledge of how fungi sense and respond to their nutritional environment, we can develop rational design strategies for protein hypersecretion. While I have elaborated on secretion of biomass degrading enzymes, the concept of (1) blinding the fungus to nutritional repression cues, (2) eliminating transcriptional repression, and (3) increasing protein translation and secretion efficiency can be applied to production of any class of enzyme involved in fungal digestive processes.
